# Long way to go: Progress of orphan drug accessibility in China from 2017 to 2022

**DOI:** 10.3389/fphar.2023.1138996

**Published:** 2023-03-08

**Authors:** Jia Liu, Yue Yu, Mingkang Zhong, Chunlai Ma, Rong Shao

**Affiliations:** ^1^ School of International Pharmaceutical Business, China Pharmaceutical University, Nanjing, China; ^2^ Department of Pharmacy, Huashan Hospital, Fudan University, Shanghai, China; ^3^ Huashan Rare Disease Center, Huashan Hospital, Fudan University, Shanghai, China

**Keywords:** orphan drug, rare disease, accessibility, availability, affordability, China

## Abstract

**Introduction:** Over 400 million patients worldwide suffer from rare diseases. Access to orphan drugs is, therefore, crucial for this population. China has been actively working on improving orphan drug accessibility in the past decades, especially since 2018 when the First National List of Rare Diseases was announced. This study aimed to evaluate the current status of orphan drug accessibility in China regarding availability, daily cost, and affordability.

**Methods:** Market availability of orphan drugs in China was based on their approval status in China up to May 2022. Information on drug availability in hospitals and the cost of each drug from 2017 to 2021 was obtained from the database of the Science and Technology Development Center of the Chinese Pharmaceutical Association. Affordability was assessed by comparing the disposable daily income per capita to the cost of the defined daily dose of each drug.

**Results:** Market availability rate was 44.3% by May 2022, and the average delay in drug approval in China compared to its orphan approval in the United States of America was 5.9 ± 6.07 years. Drug availability in hospitals showed an upward trend, with availability in tertiary hospitals significantly higher than in secondary hospitals (~20%, *p* <0.0001). The eastern area was significantly higher in availability from 2019 onwards. Fifty-eight percent of the orphan drugs were still considered to have very low availability (<30%). The national median cost of the defined daily dose across all available orphan drugs had increased to 254.97 RMB in 2021. Only 34.98% of the orphan drugs were considered affordable when compared with the national average disposable daily income in 2021, and drug affordability decreased during the past 5 years.

**Discussion:** Changes in orphan drug regulations in China have enabled progress regarding the drugs’ market availability, but the current status of drug availability at hospitals, drug cost, and affordability were not optimal. Legislation for encouraging domestic drug development and novel payment schemes for high-value drugs are essential to further improve the availability and cost burden of orphan drugs in China

## 1 Introduction

Rare or orphan diseases have a low prevalence (Franco, 2013), but are often chronically debilitating or life limiting (Dunoyer, 2011). In the United States of America (U.S.), rare diseases are defined as those diseases affecting less than 200,000 persons (prevalence <0.64‰) (Genetic and Rare Diseases Information Center, 2022). The World Health Organization (WHO) defines a rare disease based on prevalence of less than 6.5–10 in 10,000 (Franco, 2013). In China, a consensus by various organizations classifies rare diseases as those with an incidence of less than 1 in 10,000 newborns, or a prevalence of less than 1 in 10,000 adults, or patient population of less than 140,000 in total (The Joint Meeting of Chairpersons of National Rare Disease Academic Groups, 2021). Although the incidence is low for any single rare disease, approximately 7,000 rare diseases have been reported globally, with over 400 million people suffering from such diseases worldwide (Feng and Liu, 2017; Nguengang et al., 2020). A survey showed that the rare disease population in China has exceeded 20 million (Liu and Zhao, 2019).

Drugs used in rare or orphan disease diagnosis, prevention, or treatment are usually referred to as orphan drugs (Franco, 2013). On an estimate, 95% of rare diseases still lack authorized drug treatments, demonstrating a substantial unmet medical need regarding poor drug accessibility (Tambuyzer et al., 2020; Global Genes, 2022). Due to the small population size and wide variety of rare diseases, the market demand for orphan drugs is inherently low, which further increases the cost of orphan drug development (Joppi et al., 2009). It is typically very difficult for drug companies to recover the research and development costs of orphan drugs, thereby exacerbating the issue of accessibility of rare disease treatments. In 1983, the first incentivizing legislation, the Orphan Drug Act (ODA), was passed in the U.S., which not only offered hope for patients with rare diseases but also fostered a new mechanism for personalized drug and biotechnology development (Coté et al., 2010). Since then, Japan, Australia, and the European Union (EU) have legislated for orphan drugs in 1993, 1997, and 1999 respectively, which resulted in incentives for the development of such drugs (Franco, 2013). However, orphan drugs are often still unaffordable with the development costs having to be recovered across a relatively small patient population (Chan et al., 2020). For example, orphan drugs account for only 0.3% of all drugs, but 7.9% of total drug costs in the U.S.; 39% of orphan drugs cost more than $100,000 per year, nevertheless they are used to treat only 23% of patients having rare diseases (Danzon, 2018). As China has a relatively large rare disease population, the shortage of orphan drugs and the high treatment costs render the disease burden even higher in China than in other parts of the world (Gong and Jin, 2012).

Since 2007, the National Medical Products Administration (NMPA) of China has been working on the drug review and approval processes to promote fast-tracking and prioritizing review of orphan drug approvals (China Food and Drug Administration, 2018; China Food and Drug Administration, 2022). In 2018, China’s First National List of Rare Diseases, which included 121 high-prevalence and heavy-burden rare diseases, was jointly issued by five national ministries to raise public awareness and improve orphan drug accessibility. Since then, actions have been taken to establish and improve the national rare diseases registry system and disease catalogues (Song et al., 2017; He et al., 2018).

Despite progress in drug regulation, the real-world effects of policy changes on orphan drug access in China remain uninvestigated. According to the WHO definition, drug accessibility is evaluated according to the price, availability and affordability of a drug (World Health Organization and Health Action International, 2012). However, only a few studies have focused on the accessibility of orphan drugs in China. A study in 2016 showed that only 37.8%, 24.6%, and 52.4% of the orphan drugs approved in the U.S., EU, and Japan, respectively, were marketed in China, and the median availability of the 31 orphan drugs surveyed in 24 tertiary public hospitals was very low at 20.8% (Gong et al., 2016). Another study reported a delay of nine years for an orphan drug approval in China subsequent to its launch in the U.S. The study also highlighted the drugs’ low availability in hospitals (15%) and low affordability of more than half of the included drugs (Guan et al., 2019). With the exception of studies that have focused on the marketing status of orphan drugs, many studies in China have focused on affordability issues, including the national medical reimbursement system and economic burden of the selected diseases; the results were based on a specific region or a limited list of drugs surveyed (Wang and Fan, 2021; Wang and Zhao, 2021; Li and Tang, 2022; Qiao et al., 2022). It is difficult to generalize the results of studies that focused on a specific geographic area or on specific drugs, as variations in drug availability across the country are not uniform considering the size of China and the regional variability of rare disease appearance as well as economic disparities that exist.

This study aimed to comprehensively evaluate the availability, daily cost, and affordability of all orphan drugs marketed in China, as a progress update of the effect of legislation and regulations to the accessibility of orphan drugs at the national level, as at May 2022.

## 2 Methods

### 2.1 Data sources

To investigate orphan drug accessibility in China, drugs approved for an orphan indication in the U.S. Food and Drug Administration (FDA) database from 1 January 1983, to 31 May 2022, were collected as the reference drug list for this study. The domestic approval status of these drugs was investigated on the National Medical Products Administration (NMPA) website to yield a list of orphan drugs with marketing approval in China. Because the information is updated annually, information related to hospital availability and cost of the drugs, for 2017–2021, was extracted from the database of Science and Technology Development Center of Chinese Pharmaceutical Association using the non-proprietary name of each orphan drug on the above-mentioned list. Only tertiary and secondary hospitals listed in this database were included owing to the poor availability of orphan drugs in primary institutions (Gong et al., 2016). With the regional economic disparities considered, data were grouped into eastern, middle, and western areas of China, in addition to national data.

The anatomical therapeutic chemical (ATC) classification system of the WHO was used to identify the ATC code of each orphan drug for therapeutic area classification. If a drug had multiple ATC codes or no defined ATC code in the system, the ATC code (or ATC category for those not defined) was confirmed based on the therapeutic area, dosage form, and orphan disease indication of the drug. The defined daily dose (DDD) per ATC code, which is defined by WHO as the assumed average maintenance dose of a drug for its main indication per day in adults, was used in cost and affordability analyses. All information is available on the WHO ATC/DDD Index website (https://www.whocc.no/atc_ddd_index/).

### 2.2 Measures and analysis

Based on the analytical framework and methodology of the World Health Organization (WHO)/Health Action International (HAI), we analyzed the accessibility of the orphan drugs in terms of their availability, cost, and affordability.

#### 2.2.1 Availability

Orphan drug availability was measured in terms of both market- and drug-level.

In our study, the market-level availability refers to percentage of the FDA-approved orphan drugs with marketing approval in China and their respective therapeutic areas by ATC codes. The delay in marketing approvals of the studied drugs (versus launch in the U.S.) and their distribution in the year of approval were also analyzed.

Drug-level availability is the percentage of hospitals in which a particular drug can be purchased (World Health Organization and Health Action International, 2012). In our study, the drug-level availability is measured by the percentage of hospitals that reflect purchase records of any particular orphan drug. The following criteria were used to describe the availability of orphan drugs (Menn, 2006):

• Absent (0%): None of these orphan drugs were found in the institutions.
• Very low (<30%): These orphan drugs were difficult to find in the institutions.
• Low (30%–49%): These orphan drugs were not easy to find in the institutions.
• Fairly high (50%–80%): These orphan drugs were available at many of the institutions.
• High (80%): These orphan drugs were available in most institutions with good availability.

#### 2.2.2 Cost and affordability

The cost of the defined daily dose, or DDDc, is a measure of the daily cost for a patient on a maintenance dose of an orphan drug. In China, Part A drugs in the national Basic Medical Insurance (BMI) formulary are fully covered while Part B drugs are partially covered, and the level of coverage for Part B drugs varies by drug and by province. The unit procurement price was used as drug cost instead for direct comparison. The cost information for each drug was derived from the unit price reported by institutions during 2017–2021. The calculation of DDDc is as follows:

DDDc (cost of defined daily dose) = median of the unit prices of a particular drug as reported by institutions × DDD (defined daily dose).

In cases where the WHO did not provide the official DDD of a drug, the recommended daily dose for the drug’s orphan indication or main indication approved by the NMPA in adults on maintenance therapy (except drugs indicated for pediatric use) was employed. For drugs where the dose is adjusted based on weight or body surface area (BSA), the average body weight and BSA that we used in this study were calculated as 64 kg and 1.704 m^2^, respectively, for an average Chinese adult according to literature (Yu et al., 2010; Liu and Sheng, 2019; The State Council, 2020; Li and Tang, 2022).

The affordability of a certain drug is measured as the number of days a lowest-paid, unskilled government worker has to work to afford a treatment course, considering his/her daily wage (World Health Organization and Health Action International, 2012). In China, however, official information on government wages is unavailable and furthermore unrepresentative of the income level in China due to regional economic disparities. Instead, the disposable daily income *per capita* of urban and rural residents, published in the China Statistical Yearbook from 2017 to 2021, was used in our study (Yang et al., 2010; Gong et al., 2016; Guan et al., 2019; Li and Tang, 2022; National Bureau of Statistics, 2022). The DDDc of each orphan drug was compared to the national average daily disposable income as an affordability measure.

Statistical analyses were performed using R version 4.2.2. The Wilcoxon rank-sum test was used for drug-level availabilities by year and hospital type; The Kruskal-Wallis test was used for availability comparisons by economic area; and the Wilcoxon signed-rank test was used for DDDc comparisons.

## 3 Results

### 3.1 Availability

#### 3.1.1 Market-level availability

**FIGURE 1:**
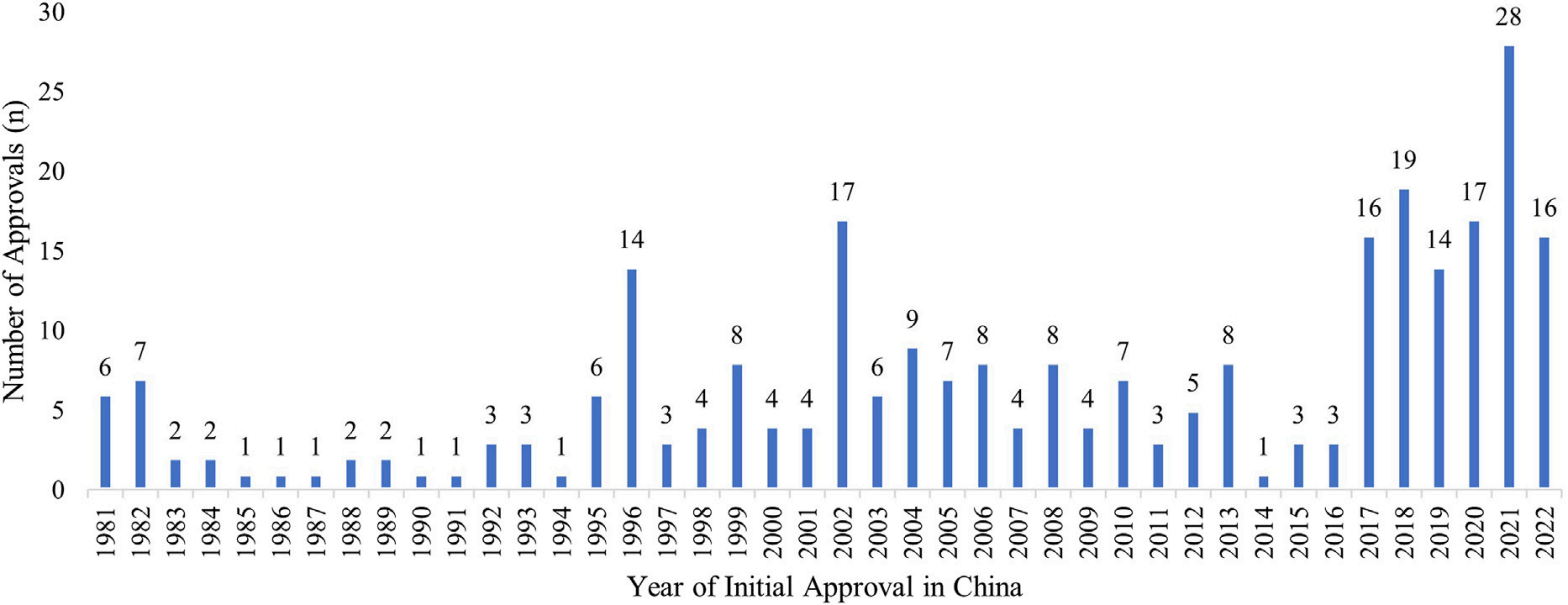
Initial Marketing Approvals in China of the FDA-approved Orphan Drugs. The distribution of initial marketing approvals in China of the 279 FDA-designated orphan drugs, up to May 2022, including drugs marketed for non-orphan indications in China prior to receiving an orphan indication by the FDA.

Of the 648 approved orphan drugs with different trade names in the FDA database from 1 January 1983, to 31 May 2022, 287 orphan drugs with unique trade names (279 unique generic names) had been approved in China. The market availability rate by trade name was therefore 44.3% (287/648). The 279 drugs with unique generic names were used for analysis in this study. Figure 1 shows the number of initial approvals by year of the 279 drugs in China. Among them, 78 were available in China before their approval for orphan indication by the FDA, and 7 were approved in China the same year of their FDA orphan designation.

A closer look at the drugs revealed a large increase in new approvals (per NMPA) from 2017 to May 2022 (Figure 1). Of these drugs, the average delay between the year the drug received an approval for orphan indication in the U.S. and its approval for marketing in China was 5.9 ± 6.07 years. The delay improved to ∼3 years in 2019, but dropped to 7.6 years in 2021, when 28 new orphan drugs were introduced into the Chinese market.

Excluding one drug with no ATC code available (prothrombin complex concentrate), “Antineoplastic and Immunomodulating Agents” (ATC Class L) was the leading drug class with 119 (42.81%) drugs approved in China (Supplementary Figure S1). Class L was the leading class across all of the study years, reaching 41.52% of all drugs approved in China in 2021 (Supplementary Table S1). Comparably, 46.07% of the drugs approved for orphan indications by the FDA from 1 January 2021, to 31 May 2022, were in ATC Class L as well (Supplementary Figure S2).

#### 3.1.2 Drug-level availability

The total number of hospitals in the Science and Technology Development Center of Chinese Pharmaceutical Association database steadily increased from 1217 (in 2017) to 1451 (in 2021). Table 1 shows the national and regional availability of marketed orphan drugs in hospitals in China across the 5-year study period. Overall, the number of drugs available and the change in availability rates showed an upward trend over time, in all hospitals and by hospital type. Tertiary hospitals had a significantly higher (*p* < 0.0001) availability of the orphan drugs compared to secondary hospitals, at around or above 20% across the eastern, middle, and western areas of China. Nationwide availability across all hospitals, however, was not significantly different each year (*p* > 0.4). Comparison of the drug availabilities among the three economic areas for each year showed significant differences in 2019 (*p* = 0.024), 2020 (*p* = 0.014), and 2021 (*p* = 0.003), with the eastern area being the highest.

**TABLE 1:** Median availability of orphan drugs in hospitals in China from 2017 to 2021 (%).

Area	Year	n^a^	All hospitals		Tertiary hospitals		Secondary hospitals		p^c^
			Availability	Median change^b^	Availability	Median change	Availability	Median change	
Nationwide	2017	162	18.78		23.91		6.34		0.00
	2018	178	17.31	−0.58	21.15	−0.53	5.48	−0.17	0.00
	2019	190	18.65	0.00	24.15	0.07	6.05	0.09	0.00
	2020	208	18.17	0.25	23.08	0.36	6.03	0.21	0.00
	2021	223	17.99	0.06	23.08	0.24	5.62	0.03	0.00
Eastern Area	2017	162	22.60		27.52		7.88		0.00
	2018	178	19.46	−0.34	23.64	−0.05	7.74	−0.04	0.00
	2019	190	24.15	0.50	27.27	0.64	8.18	0.56	0.00
	2020	208	23.10	0.24	27.13	0.39	8.28	0.00	0.00
	2021	223	22.53	0.31	27.73	0.44	7.91	0.00	0.00
Middle Area	2017	162	15.66		19.83		4.17		0.00
	2018	178	14.59	−0.75	18.18	−0.94	3.90	−0.11	0.00
	2019	190	14.06	−0.13	18.92	−0.03	4.07	0.00	0.00
	2020	208	13.65	0.00	18.53	0.00	4.17	0.00	0.00
	2021	223	13.77	−0.16	19.14	0.02	3.47	−0.04	0.00
Western Area	2017	162	15.92		19.81		5.80		0.00
	2018	178	15.00	0.22	18.67	−0.48	5.41	0.00	0.00
	2019	190	16.54	0.04	20.11	−0.12	5.49	0.00	0.00
	2020	208	16.61	0.73	21.43	0.67	4.88	1.22	0.00
	2021	223	17.02	0.33	21.21	0.00	5.95	1.05	0.00

^a^ n = number of orphan drugs with data.

^b^ Median change is defined as the median of the annual changes or differences in the drug-specific availability in each hospital.

^c^ *p* value of Wilcoxon rank-sum test for the difference of orphan drugs’ median availability at drug level between tertiary hospitals and secondary hospitals each year.

**FIGURE 2:**
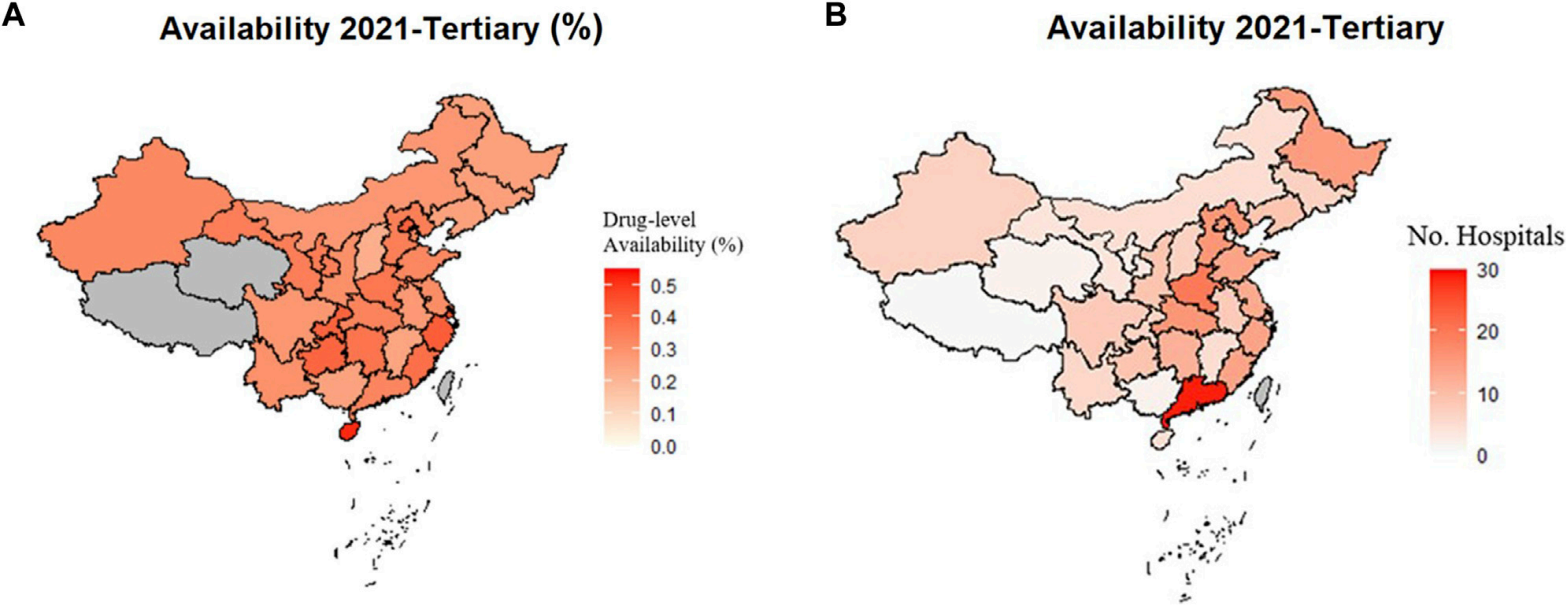
Availability of Orphan Drugs in Tertiary Hospitals in 2021 by Province. Greyed areas represent the excluded results due to data not collected or total number of hospitals under five. **(A)** Availability at drug-level in tertiary hospitals by percentage in each province in 2021. **(B)** Availability at drug-level in tertiary hospitals by number of hospitals in each province in 2021.

Considering the tertiary hospitals only, province-specific results showed that Hainan (52.01%), Shanghai (45.62%), and Zhejiang (43.03%) were the highest in terms of percentage of hospitals having orphan drug availability. By hospital numbers, Guangdong was far ahead with a prevailing average of 29 tertiary hospitals offering specific orphan drugs, followed by Henan with 20 hospitals. All of the above-mentioned provinces are in the eastern area except Henan province which is in the middle area. The province-specific availability data are shown in Figure 2.

Although orphan drug availability increased over time, more than half (58%) of the drugs were still considered very low (<30%) in availability, even in tertiary hospitals (Supplementary Table S2). Despite an increase in the number of drugs approved in each availability category over the study period, none of the drugs were considered high (>80%) in availability in 2021.

### 3.2 Cost and affordability

A total of 180 (64.5%) of the 279 drugs were covered by BMI in 2021, and only 31 (11.1%) of them had full coverage as Part A drugs. The median DDDc varied over the study period and across the different economic areas, but generally showed an increasing trend (Tables 2, 3). The national median DDDc of orphan drugs increased from 118.83 RMB to 254.97 RMB during the study period, while the interquartile ranges (IQRs) grew simultaneously. The middle area had the lowest DDDc among all areas based on the drug cost information available. The DDDc barely changed over the years of the study period in any of the three economic areas, but a slight yet statistically significant reduction in the median change of DDDc was observed in the nationwide data.

**TABLE 2:** Change in cost of defined daily dose (DDDc) nationwide of orphan drugs in China (RMB).

Year	n^a^	DDDc	IQR^b^	Median change^c,d^	IQR
2017	144	118.83	575.46		
2018	152	137.05	576.90	−0.19*	13.73
2019	165	178.64	704.35	−0.05*	6.97
2020	183	225.37	873.85	0.00*	3.61
2021	203	254.97	1121.82	−0.10*	15.03

^a^ n = number of orphan drugs with procurement price data.

^b^ IQR: interquartile range, equals to the difference between 75th and 25th percentiles.

^c^ Median change is defined as the median of the annual changes or differences in the drug-specific defined daily cost (DDDc) between years.

^d^ *p* value of Wilcoxon signed-rank test for the difference of orphan drugs’ median DDDc, within the area each year. *for *p*-value <0.0001.

**TABLE 3:** Change in cost of defined daily dose (DDDc) by economic area of orphan drugs in China (RMB).

	Eastern area					Middle area					Western area				
Year	n^a^	DDDc	IQR^b^	Median change^c^	IQR	n	DDDc	IQR	Median change	IQR	n	DDDc	IQR	Median change	IQR
2017	143	127.87	584.81			131	99.66	464.95			125	104.85	469.53		
2018	150	135.99	625.43	0.00	2.85	144	138.34	610.83	−0.19	23.56	142	131.39	575.49	−0.30	15.69
2019	164	170.34	779.77	0.00	3.03	158	138.34	610.83	0.00	2.40	157	166.17	643.96	0.00	2.16
2020	181	204.67	872.43	0.00	2.34	169	187.15	681.82	0.00	0.00	176	208.42	843.66	0.00	1.35
2021	194	240.06	924.53	0.00	0.87	190	215.07	604.81	−0.01	12.80	193	246.83	955.15	0.00	3.82

^a^ n = number of orphan drugs with procurement price data.

^b^ IQR: interquartile range, equals to the difference between 75th and 25th percentiles.

^c^ Median change is defined as the median of the annual changes or differences in the drug-specific defined daily cost (DDDc) between years.

The Wilcoxon signed-rank test compared the paired DDDc between years (where there existed drug cost information for both years) and showed significance (all *p* < 0.0001) in DDDc changes.

**FIGURE 3:**
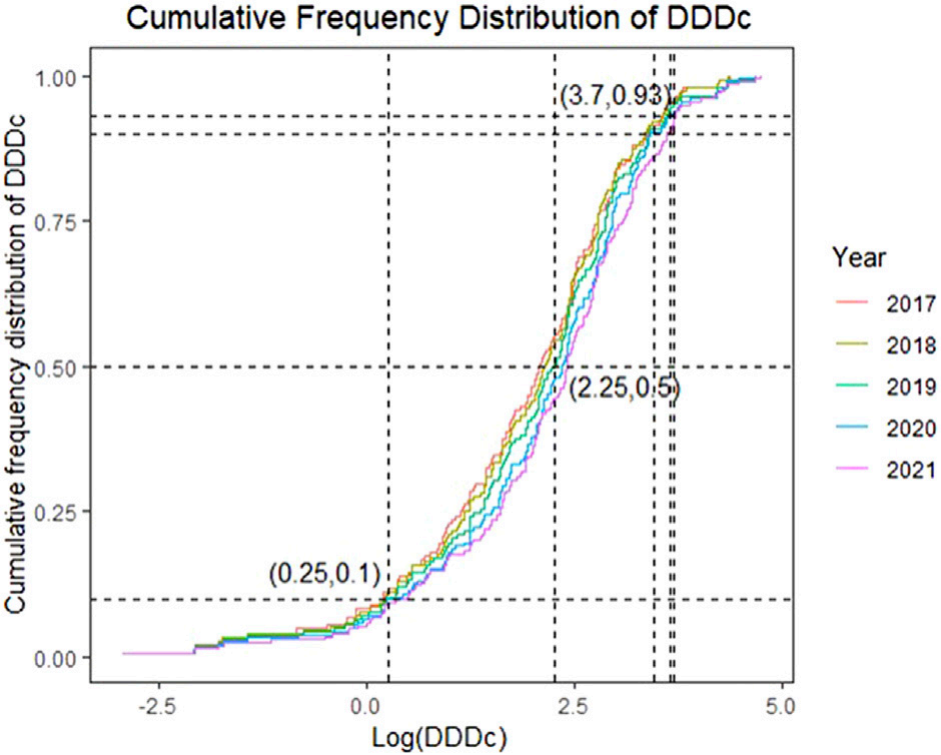
Cumulative Frequency Distribution of the Cost of Defined Daily Dose (DDDc) from 2017 to 2021 (in log scale).

Figure 3 presents the cumulative distribution of DDDc over time on a logarithmic scale. A cumulative frequency of 0.5 was at log value of 2.25, which means half of the drugs were above 178 RMB for 1-day treatment; drugs with DDDc in the lowest 10% (log value of 0.25, approx. 2 RMB) did not vary significantly between years, whereas drugs with DDDc in the highest 10% showed a substantial increase from over 2,800 RMB (log value 3.44) during 2017–2020 to 4,500 RMB (log value 3.65) in 2021. The DDDc of drugs in the top 7% (cumulative frequency 0.93) were close to one another over the 5-year study period at above a log value 3.7 or 5,000 RMB.

Less than half of the orphan drugs were considered affordable when comparing their DDDc with the average disposable daily income of urban and rural residents during 2017–2021 (Supplementary Table S3). The results showed a downward trend in affordability over the years (Supplementary Figure S3), with urban residents having better affordability than the rural residents do. On average, only 34.98% of the orphan drugs had a DDDc no more than the daily income of an average resident in 2021. The affordability for urban residents improved from 41.08% in 2020% to 41.87% in 2021, increasing the affordability gap between urban and rural residents. Overall, orphan drug affordability decreased, whereas DDDc increased for all residents.

## 4 Discussion

This study assessed orphan drug accessibility in China at the national and regional levels in all hospitals nationwide. Our results reflect the achievements in drug marketing approvals, with highlighting the issues with availability and affordability of orphan drugs in China. To our knowledge, this study may be the only update so far at the national level to the previous study findings of the period 2011–2017, with inclusion of all approved drugs and hospitals that treat rare diseases (Guan et al., 2019). As the status of orphan drug accessibility in China has been evolving since 2017, the results of this study are expected to provide practical information to policymakers and stakeholders for their consideration in the reinforcing of orphan drug policies.

As expected, additional drugs have been approved in China since 2017: a total of 279 different generic names in this study as compared to the 150 in the previous one (Guan et al., 2019). Comparing with the literature results at 2017, the market availability rate at 2021 improved from 28.8% to 44.3% and the average delay in approval shortened from 9.0 to 5.9 years (Guan et al., 2019). A key factor in the surge in market availability was the regulatory changes: a policy was announced in 2016 to expedite the orphan drug review and market approval processes (China Food and Drug Administration, 2016); then in 2018, China’s first List of Rare Diseases (CLRD) was released, serving as the first official national definition and focus list of rare diseases (National Health Commission Ministry of Science and Technology, 2018). Since then, the import of novel drugs into China has increased markedly, based on an urgent unmet medical need in the domestic market. In 2021, the year in which new drug approvals peaked, the official Technical Guidance for the Research and Development of Orphan Drugs was released by the Center for Drug Evaluation (Center for Drug Evaluation NMPA, 2021), which further raised awareness and market interest in rare diseases and orphan drugs.

Nearly half of the orphan drugs fall into the therapeutic category of antineoplastic and immunomodulating agents (Supplementary Figure S1, S2), consistent with the therapeutic area distribution of FDA-approved drugs with orphan designation (Tambuyzer et al., 2020; Wang and Fan, 2021). Although many Class L drugs are immunotherapies that can be used to treat a range of rare diseases besides the cancers they are designated for, the FDA tends to more easily approve orphan drugs that have cancer designations. This is especially true of drugs for those cancers that are relatively more prevalent, and FDA does not require significantly greater benefits to be demonstrated for a drug to be approved for an indication that already has available treatments (Vokinger and Kesselheim, 2019). Cancer drugs may conceivably receive approval more easily than those for non-cancer designations and generate revenues relatively sooner, yielding a quicker return on investment (Light and Lexchin, 2015), which may explain the market enthusiasm for cancer drugs. As a result, the market efforts were leaned toward the less-rare diseases (Gamba et al., 2021). However, policy-making should rather be guided by patient demands. An analysis of the Orphanet database showed that 71.9% of rare diseases are genetic and 69.9% are exclusively onset at pediatric stage (Nguengang Wakap et al., 2020). A Chinese study reported that 102 types of the rare diseases listed in the CLRD accounted for 54,468 hospitalizations during 2014–2015, and nearly one-third of the patients were under 14 years of age (Shi et al., 2018). Correspondingly, the CLRD has indicated a priority focus on rare diseases, as most of the 121 rare diseases in 2018 CLRD were genetic and metabolic, and many were onset at the pediatric stage with a resultant heavy disease burden carried into adulthood. Though the prevalence and definition of rare diseases are different globally due to genetic and environmental factors, the numerous immunotherapy products on the market in China should attract attention because they have been used for treating an increasing number of the 121 rare diseases. Policy support in designation of existing immunomodulating agents for orphan diseases is highly desired, and domestic research and development (R&D) should be encouraged for faster market access and lower economic burden to patients (Cheng and Xie, 2017).

The drug-level availability was rather static over the past 5 years nationwide and in the eastern, middle, and western areas, whilst the number of drug approvals increased, probably because the new orphan drugs went to the same hospitals designated for rare disease treatment every year. Availability in tertiary hospitals was significantly higher than in secondary hospitals, which is consistent with their respective medical capabilities in rare disease diagnosis and treatment (Li et al., 2021). Provinces in the eastern area are higher in hospital access to orphan drugs as visualized in Figure 2. Availability of orphan drugs are affected by access to proper diagnostics and treatment, local medicine expenditure of the government, and socioeconomic status of patients (Logviss et al., 2014; Detiček et al., 2018; Gahl et al., 2021). The higher availability rates in the eastern area likely reflect better orphan drug access and more condensed distribution of the designated hospitals (Shi et al., 2018). Since the eastern area is more economically developed, more medical resources are attracted that benefit rare disease education to both the healthcare providers and the patients. Improved rare disease advocacy can help prevent misdiagnosis and increase patients’ chances of receiving professional care. Availability of orphan drugs is hence improved through increased treatment demands.

Among provinces in the eastern area, Hainan, the island on the bottom left of the map, had the highest percentage of orphan drug availability in tertiary hospitals (Figure 2A). The Hainan Free Trade Port Law has given Hainan autonomy in policymaking in many areas, including the import of foreign medicines and sanitary equipment (Qi et al., 2021). Many novel, high-value drugs were available quickly owing to the rapid new drug approval process in Hainan. In contrast, the availability by number of hospitals in Guangdong was almost double that of the other provinces (Figure 2B). Although Guangdong was not high in orphan drug availability by percentage due to the high number of total hospitals, the sheer number of hospitals with orphan drug access translated to easier drug acquisition for patients in Guangdong.

Overall, orphan drug availability in China remains low. More than half of the drugs were considered to have very low (<30%) availability, and those with high availabilities are often drugs that are commonly used for non-orphan indications, such as octreotide and methylene blue. Possible reasons for the differences in drug-level availability include: 1) Delay in market response or hospital procurement process, like most of the novel antineoplastic/immunomodulating agents. These drugs generally increase rapidly in hospital availability. The approvals of these new drugs each year contribute to the decreased availability rates due to their low hospital coverage initially. 2) Degree of rare disease awareness and knowledge among medical professionals, as well as the level of education and social status of the target patient population. As previously mentioned, these two factors affected the treatment demands thus the availability in hospital. 3) Different patient sizes and market awareness. Orphan drugs targeting non-oncology diseases might not grow as much in availability as the cancer drugs do. Emicizumab received NMPA authorization for hemophilia A in 2018, but its availability remained very low (0.40% in 2021). Only one hospital showed purchase record in 2018 in the entire country, and in 2021 it slightly improved to four hospitals, each from a different province. Fingolimod was imported in 2019 for multiple sclerosis, but no hospital purchase records were found during the study period. More attention needs to be paid to improve real world utilization of these barely available drugs. A wider spread of orphan drugs in different hospitals and a higher number of designated hospitals for rare disease diagnosis and treatment may be effective in improving the utilization and hospital coverage of orphan drugs.

The cost of orphan drugs in China has generally increased as measured by the median DDDc, but the changes in DDDc over the years have been negative or close to zero, indicating a slight downward trend in the cost of each particular drug. The year-on-year changes in DDDc nationwide were not significantly different per the Wilcoxon rank-sum test, however the Wilcoxon signed-rank test showed a significance in the annual changes. Together with the DDDc results, the statistical analyses inferred that a significant portion of the drugs had insignificant variation in their costs over the study years. The differences between the results of 2017 compared with 2021 were also insignificant; it was thought reasonable for drug costs to be maintained or marginally decreasing each year over just 5 years (Guan et al., 2019). The expanding IQRs indicated that the increase in median DDDc each year was probably caused by the newly approved drugs (Qiao et al., 2022), which are typically highly expensive and contribute to the widening range of the DDDc overall. The number of drugs analyzed in each economic area differed according to the cost information available; therefore, regional comparisons could not be performed.

The cumulative distribution curves of DDDc for 2017–2021 (Figure 3) almost merged in the lower 10% and top 7% but deviated in the middle 83%, which means the cost of the cheapest and the most expensive drugs did not vary much over the years, but the cost distribution of the majority of drugs increased as new drugs were added. Interestingly, the sharp increase in the top 10% DDDc of the drugs in 2021 was in accordance with the surge in drug approvals that year, mostly contributed by the high-value drugs. As DDDc increased, affordability decreased, regardless of the resident type (urban or rural). The higher income levels of urban residents translates to their higher affordability versus rural residents; however, less than half of the drugs could be afforded for either resident type, which corroborated the results of the 2011–2017 study (Guan et al., 2019).

Drug cost and affordability have not improved since 2017, and the reasons may be threefold: 1) Drug costs may have reduced in some provinces because of volume-based procurement, yet a change may not be noticeable at the national level (across 22 provinces) and over five years only; 2) As new drugs enter the Chinese market, their typically high prices substantially influence the affordability assessment as represented by the median DDDc; 3) The lack of incentives for the domestic R&D of orphan drugs, patent protection of imported branded products, and limited competition within orphan drug indications all allow the high costs of the imported orphan drugs to be maintained.

China has long been behind on rare disease legislation. The stricter definition of rare disease compared to other countries in addition to the limited number of diseases included in CLRD leaves the needs of many more rare disease patients in China to be fulfilled. Despite the amount of work done under the Healthy China 2030 Action Plan, there has not been a national plan or official organization dedicated to rare diseases in China. A national plan specific for rare diseases can function as a regulatory framework to promote downstream policies that improve orphan drug accessibility. Incentivizing policies can largely affect orphan drug access in multiple ways, from treatment awareness to indication approvals. Legislation to further the recognition of rare disease is necessary to guide the resources that ultimately improve the disease awareness, ability of diagnosis and treatment, and orphan drug availability (Koçkaya et al., 2014; Murakami and Narukawa, 2016; Dharssi et al., 2017; Gamba et al., 2021).

Overall, positive progress has been made in market-level availability in recent years, but drug-level availability and affordability remain unfavorable. Now that policy reform in rare diseases has yielded initial effects, further measures need to be taken regards legislative and policy change and implementation that focuses on drug-level availability and affordability improvement. First, policies for improving hospital procurement and medical use should be accelerated. While the number of drug approvals has been increasing, the actual use or sales of new orphan drugs has not increase concomitantly. Requirements that limit pharmacy expenditures and the total drug costs of medical orders or prescriptions are obstacles to the use of high-value orphan drugs in hospitals (The State Council of the People’s Republic of China, 2022; Guan et al., 2016). Second, it is important to enhance the nationwide distribution of hospitals that can provide orphan drugs. The high cost and low demand of orphan drugs creates warehousing and drug turnover barriers, meaning that non-designated hospitals have little interest nor ability to purchase orphan drugs. Policy guidance to relocate medical and financial resources would alleviate the regional disparities of orphan drug access. By raising the diagnostic ability of providers and disease awareness of patients, treatment demands will be increased and the drug availability will be improved. Third, cost-lowering strategies should be encouraged. Policies for incentivizing and encouraging local R&D to invent new drug entities can play a critical enabling role (Brown and Wobst, 2021). Higher availability and utilization of high-quality domestically-developed products could help address cost issues. Regarding cost coverage, new payment mechanisms or tailored reimbursement management for orphan drugs are necessary to balance the needs of patients with the burden of drug procurement on the national health security system. A multi-criteria payment model that fits China’s situation could be developed using international experiences with orphan drug payment models, such as third-party commercial health plans and outcomes-based payment schemes (Schey et al., 2011; de Andrés-Nogales et al., 2021; Zelei et al., 2021). National negotiation of prices or volume-based procurement may not be the best solution for long-term reimbursement of costly orphan drugs. More parties need to be involved in cost-sharing of these high-value products.

This study had certain limitations. First, FDA-approved orphan drugs were used as the sole reference; drugs approved in other countries were excluded from this study. Given that the FDA approves the highest number of orphan drugs globally (Korchagina et al., 2015), the impact on our results should be limited. Second, our hospital datasets were obtained exclusively from the database of the Science and Technology Development Center of Chinese Pharmaceutical Association. The information on drug availability and procurement prices was limited by what data was available in the database, and dependent on the network membership status and annual data reporting of each hospital (which may not reflect the actual accessibility). Third, medical insurance coverage and patient assistance programs were not considered, hence affordability may have been underestimated. However, reimbursement schemes vary widely by individual, program, and area, thus making the impact hard to quantify.

## 5 Conclusion

This study revealed the overall low accessibility of orphan drugs in China currently. Although legislation and its effects take time, achievements have non-etheless been made in marketing approvals. However, unsatisfactory drug availability in hospitals, high costs of treatment, and low affordability are a justification for additional, more specific, progressive policies that can enable alleviation of this situation. A high number of new orphan drug approvals are meaningless if the drugs are not available or affordable. With decreasing availability and growing drug prices across the years of the study period, incentivizing policies are urgently required to encourage domestic new drug R&D. Appropriate legislation could pave the way for hospital procurement and innovative payment schemes. As public awareness of rare diseases continues to rise in China, continuous progress in the accessibility of orphan drugs is foreseeable for the near future.

## Data Availability

The original contributions presented in the study are included in the article/Supplementary Material, further inquiries can be directed to the corresponding authors.
