# Supplementary material for: Long way to go: Progress of orphan drug accessibility in China from 2017 to 2022
Source: Front Pharmacol. 2023 Mar 8;14:1138996. doi: 10.3389/fphar.2023.1138996 (PMC10031016; doi:10.3389/fphar.2023.1138996)
Supplement: Supplementary file 1 [file DataSheet1.docx]

Supplementary Material

**Long way to go: progress of orphan drug accessibility in China from 2017 to 2022**

**Jia Liu, Yue Yu, Mingkang Zhong, Chunlai Ma*, Rong Shao^*^**

*** Correspondence:** Chunlai Ma, [chunlaima@126.com](mailto:chunlaima@126.com); Rong Shao, [shaorong118@163.com](mailto:shaorong118@163.com)

# Supplementary Figures


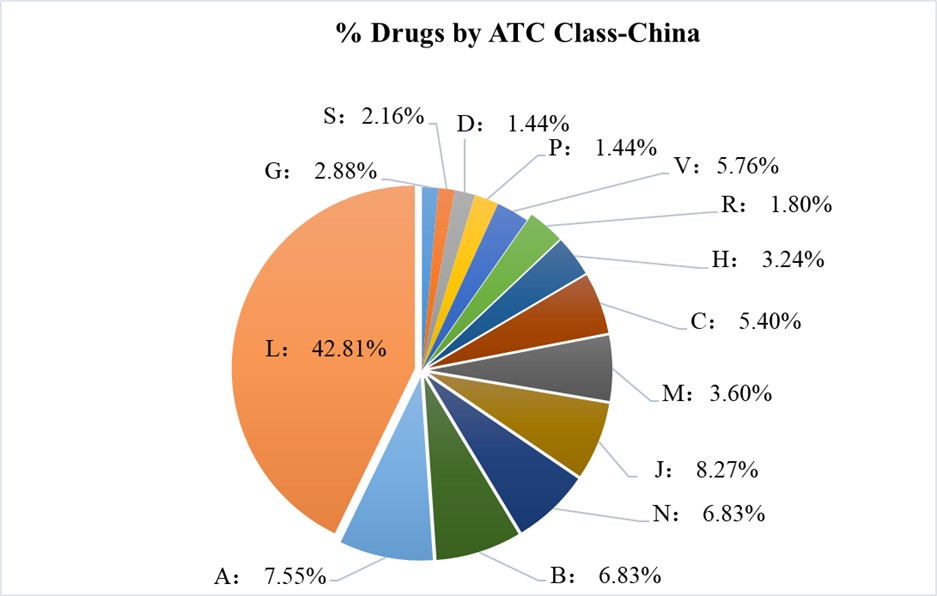


**Figure S1.** ATC Classifications of Orphan Drugs Marketed in China with FDA approvals by May 2022


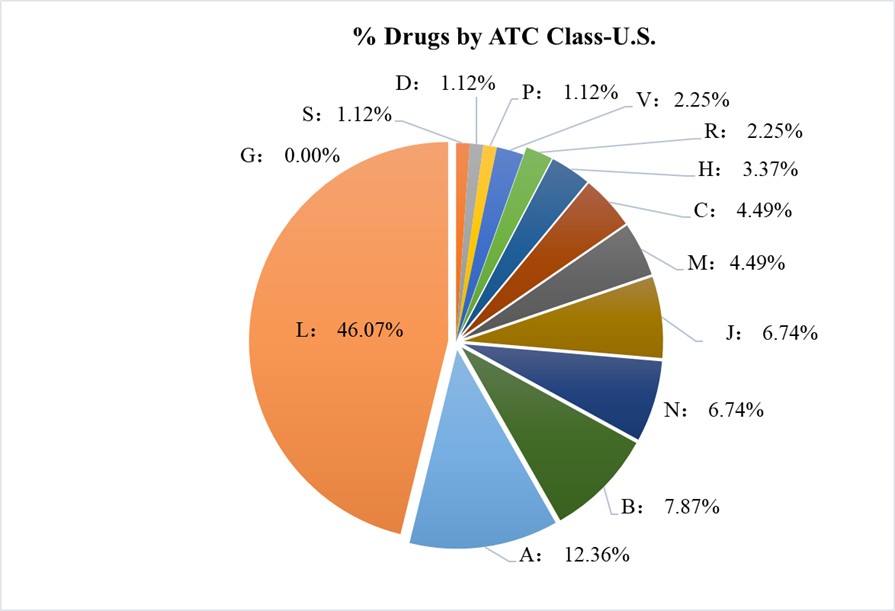


**Figure S2.** ATC Classifications of Orphan Drugs Approved by U.S. FDA from Jan 2021 to May 2022


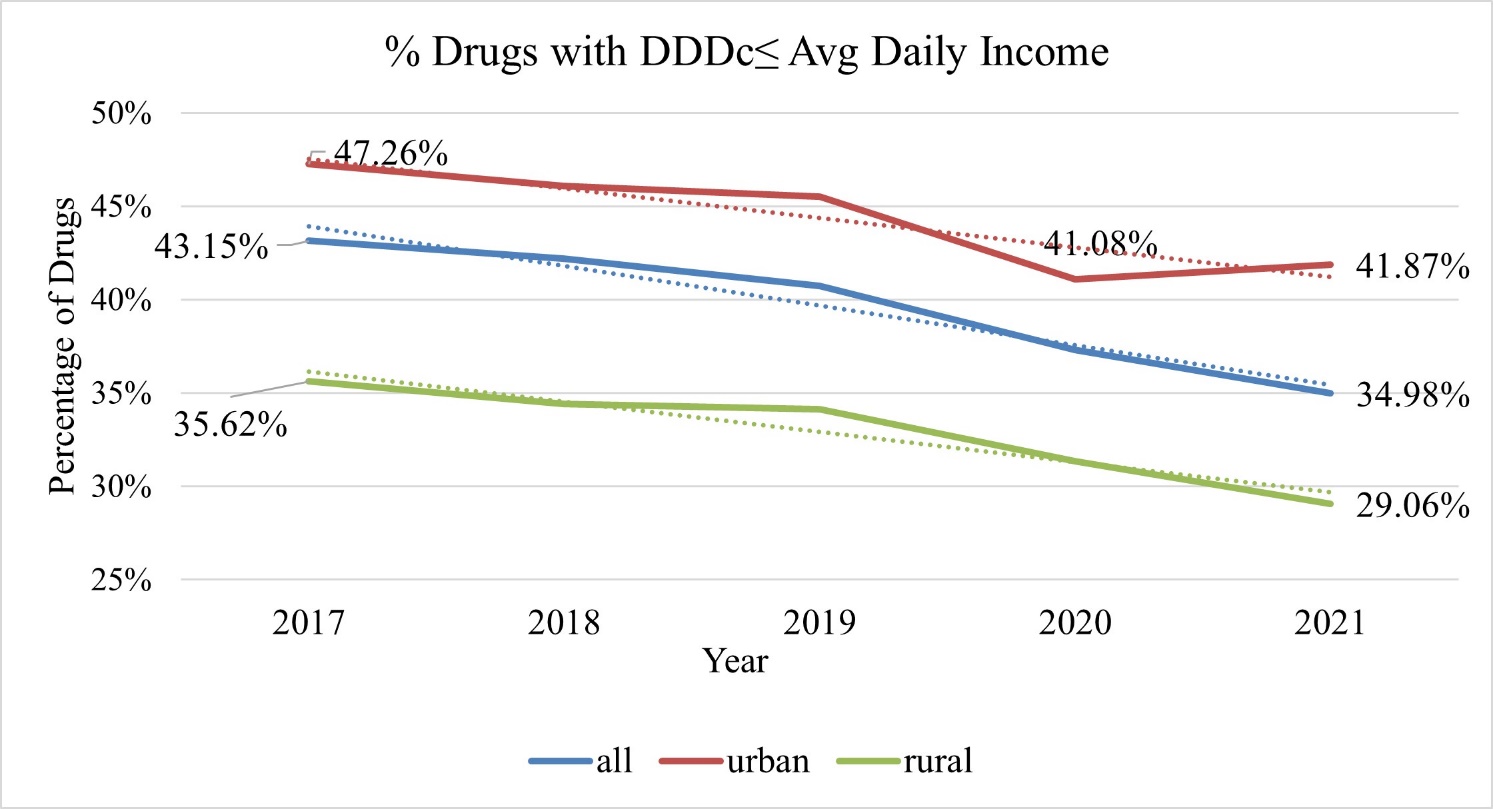


**Figure S3**. Affordability of drugs measured by comparing drug DDDc and average daily income.

# Supplementary Tables

| Year | 2017 | | 2018 | | 2019 | | 2020 | | 2021 | |
| --- | --- | --- | --- | --- | --- | --- | --- | --- | --- | --- |
| ATC Class | Drugs  (n) | Percentage | Drugs  (n) | Percentage | Drugs  (n) | Percentage | Drugs  (n) | Percentage | Drugs  (n) | Percentage |
| A | 13 | 6.91% | 13 | 6.25% | 15 | 6.70% | 17 | 6.97% | 20 | 7.22% |
| B | 11 | 5.85% | 13 | 6.25% | 13 | 5.80% | 16 | 6.56% | 19 | 6.86% |
| C | 13 | 6.91% | 14 | 6.73% | 15 | 6.70% | 15 | 6.15% | 16 | 5.78% |
| D | 2 | 1.06% | 3 | 1.44% | 3 | 1.34% | 4 | 1.64% | 4 | 1.44% |
| G | 6 | 3.19% | 7 | 3.37% | 7 | 3.13% | 7 | 2.87% | 7 | 2.53% |
| H | 9 | 4.79% | 10 | 4.81% | 10 | 4.46% | 10 | 4.10% | 10 | 3.61% |
| J | 17 | 9.04% | 19 | 9.13% | 21 | 9.38% | 22 | 9.02% | 25 | 9.03% |
| L | 67 | 35.64% | 77 | 37.02% | 87 | 38.84% | 96 | 39.34% | 115 | 41.52% |
| M | 7 | 3.72% | 7 | 3.37% | 8 | 3.57% | 8 | 3.28% | 10 | 3.61% |
| N | 16 | 8.51% | 16 | 7.69% | 16 | 7.14% | 19 | 7.79% | 20 | 7.22% |
| P | 4 | 2.13% | 4 | 1.92% | 4 | 1.79% | 4 | 1.64% | 4 | 1.44% |
| R | 3 | 1.60% | 3 | 1.44% | 3 | 1.34% | 3 | 1.23% | 4 | 1.44% |
| S | 6 | 3.19% | 6 | 2.88% | 6 | 2.68% | 7 | 2.87% | 7 | 2.53% |
| V | 14 | 7.45% | 16 | 7.69% | 16 | 7.14% | 16 | 6.56% | 16 | 5.78% |
| Total | 188 |  | 208 |  | 224 |  | 244 |  | 277 |  |

**Table S1:** ATC Classifications of Orphan Drugs approved in China from 2017 to 2021 (cumulative).

**Table S2**. Category of Orphan Drug Availability by Drug-level from 2017-2021 in China. **(A)** Drug availability in all hospitals. **(B)** Drug availability in tertiary hospitals.

**(A)** Drug availability in all hospitals

| Category^a^ | Year | n^b^ | Category | Year | n | Category | Year | n | Category | Year | n |
| --- | --- | --- | --- | --- | --- | --- | --- | --- | --- | --- | --- |
| 1-29% | 2017 | 97 | 30-49% | 2017 | 31 | 50-80% | 2017 | 29 | 81-100% | 2017 | 5 |
|  | 2018 | 112 |  | 2018 | 33 |  | 2018 | 31 |  | 2018 | 2 |
|  | 2019 | 120 |  | 2019 | 38 |  | 2019 | 31 |  | 2019 | 1 |
|  | 2020 | 134 |  | 2020 | 41 |  | 2020 | 33 |  | 2020 | 0 |
|  | 2021 | 148 |  | 2021 | 43 |  | 2021 | 32 |  | 2021 | 0 |

**(B)** Drug availability in tertiary hospitals

| Year | Category^a^ | n^b^ |  | Year | Category | n |  | Year | Category | n |  | Year | Category | n |  | Year | Category | n |
| --- | --- | --- | --- | --- | --- | --- | --- | --- | --- | --- | --- | --- | --- | --- | --- | --- | --- | --- |
| 2017 | 0-29 | 93 |  | 2018 | 0-29 | 107 |  | 2019 | 0-29 | 107 |  | 2020 | 0-29 | 120 |  | 2021 | 0-29 | 131 |
|  | 30-49 | 27 |  |  | 30-49 | 31 |  |  | 30-49 | 41 |  |  | 30-49 | 44 |  |  | 30-49 | 48 |
|  | 50-80 | 36 |  |  | 50-80 | 36 |  |  | 50-80 | 40 |  |  | 50-80 | 43 |  |  | 50-80 | 44 |
|  | 81-100 | 6 |  |  | 81-100 | 4 |  |  | 81-100 | 2 |  |  | 81-100 | 1 |  |  | 81-100 | 0 |
| Total |  | 162 |  |  |  | 178 |  |  |  | 190 |  |  |  | 208 |  |  |  | 223 |

^a^Category refers to the percentage level of availability according to the following criteria: absent (0%): none of these orphan drugs were found in the institutions; very low (<30%): these orphan drugs were difficult to find in the institutions; low (30–49%): these orphan drugs were not easy to find in the institutions; fairly high (50–80%): these orphan drugs were available at many of the institutions; high (80%): these orphan drugs were available in most institutions with good availability.

^b^n is the number of drugs fall into the specific level of availability that year.

**Table S3.** Affordability of orphan drugs measured by percentages with DDDc≤average daily income by urban, rural, or all residents in China.

| Year | Type of Residents | DDDc ≤ Avg. Daily Income | DDDc＞Avg. Daily Income | % Drugs with DDDc ≤Avg. Daily Income |
| --- | --- | --- | --- | --- |
| 2017 | All | 63 | 83 | 43.15% |
|  | Urban | 69 | 77 | 47.26% |
|  | Rural | 52 | 94 | 35.62% |
| 2018 | All | 65 | 89 | 42.21% |
|  | Urban | 71 | 83 | 46.10% |
|  | Rural | 53 | 101 | 34.42% |
| 2019 | All | 68 | 99 | 40.72% |
|  | Urban | 76 | 91 | 45.51% |
|  | Rural | 57 | 110 | 34.13% |
| 2020 | All | 69 | 116 | 37.30% |
|  | Urban | 76 | 109 | 41.08% |
|  | Rural | 58 | 127 | 31.35% |
| 2021 | All | 71 | 132 | 34.98% |
|  | Urban | 85 | 118 | 41.87% |
|  | Rural | 59 | 114 | 34.10% |
